# Increased 10-year cardiovascular disease risk in depressed patients with coexisting subclinical hypothyroidism

**DOI:** 10.3389/fpsyt.2023.1185782

**Published:** 2023-07-04

**Authors:** Shuai Zhao, Boyu Zhang, Yuqin Han, Jianjun Guan, Wenmei Fang, Hongqin Zhang, Anzhen Wang

**Affiliations:** ^1^Department of Psychiatry, The Affiliated Psychological Hospital of Anhui Medical University, Hefei, China; ^2^Hefei Fourth People’s Hospital, Hefei, China; ^3^Anhui Mental Health Center, Hefei, China; ^4^Anhui Clinical Research Center for Mental Disorders, Hefei, China

**Keywords:** subclinical hypothyroidism, depression, cardiovascular disease risk, female, comorbidity

## Abstract

**Purpose:**

The prevalence of depressive disorder (DD) and subclinical hypothyroidism (SH) was almost twofold higher in women compared with men, both of which are confirmed to be related to cardiovascular disease (CVD) risk. The current study aimed to identify the prevalence of CVD risk factors and evaluate the 10-year CVD risk in female depressed patients with and without comorbid SH.

**Methods:**

We recruited 1744 female inpatients with a diagnosis of DD. Venous blood samples were taken from all patients for lipid and thyroid hormones. Framingham Risk Score (FRS) was used to estimate the 10-year CVD risk.

**Results:**

Female depressed patients with SH had increased BMI, higher Hamilton Anxiety Scale (HAMA) scores, higher LDL-C, TC, UA, and a higher 10-year CVD risk than euthyroid DD groups. Serum TSH levels and HAMA scores were critical predictive variables for 10-year CVD risk in female depressed patients with comorbid SH.

**Conclusion:**

Our study suggests that female depressed patients with SH have a high 10-year CVD risk. Serum TSH levels and HAMA scores may be helpful to predict cardiovascular risk in female patients with SH. The increased CVD risk in female depressed patients with comorbid SH requires more attention from researchers and clinicians.

## 1. Introduction

Depression is a prevalent, costly, and life-threatening mental disorder whose mechanism has not been fully understood yet, with a lifetime prevalence of 6.8% among adults in China (1). Previous studies have reported potential links between depression and cardiovascular disease (CVD) (2–4). A complex relationship between depression and CVD is a chronic condition significantly affecting depressed individuals’ health. Although substantial advances have been made in understanding and treating these disorders, comorbid CVD with depression is prone to have a worse prognosis and high health burden than a depression or CVD alone (5). The World Health Organization (WHO) reports that depression and CVD independently and concurrently lead to enormous healthcare costs and represent the most common causes of disability in high-income countries (6). Despite the established association, the underlying The prevalence of depressive disorder (DD) and subclinical hypothyroidism (SH) was almost twofold higher in women compared with men, both of which are confirmed to be related to cardiovascular disease (CVD) risk. The current study aimed to identify the prevalence of CVD risk factors and evaluate the 10-year CVD risk in female depressed patients with and without comorbid SH. pathophysiological mechanisms linking CVD and depression are unclear. Nevertheless, inflammation, genetic, hormonal, and psychosocial factors are hypothesized to play a role (7, 8). A review by Corona et al. (9) recently provides a comprehensive overview of the clinically relevant relationship between the thyroid and heart, highlighting the cardiovascular relation with overt and subclinical thyroid dysfunctions.

Subclinical hypothyroidism (SHypo) represents an early form of thyroid dysfunction and is defined by elevated serum thyroid-stimulating hormone (TSH), normal free thyroxine (FT4), and free triiodothyronine (FT3) concentrations (10). The incidence of SHypo varies among populations, ranging from 3 to 15%, with increasing age, low iodine intake, and those with a family history of thyroid disease (11). The prevalence of SHypo is higher in women (6–10%) than in men (11, 12). SHypo and CVD are closely associated and have an adverse impact on each other (13, 14). Recently, a retrospective cohort study by Kosuke et al. (2) found that CVD mediated the associations of SHypo with all mortality. Notably, one recent study found that the prevalence of SHypo in patients with depression was 60% (15). Suboptimal management of either disease may further increase the burden of CVD. Therefore, *awareness* of the cardiovascular risk for depressed patients with comorbid SHypo should be promoted.

The Framingham risk score (FRS) is a tool for estimating 10-year CVD risk, widely applied among patients with psychiatric disorders (16). CVD risk refers to the likelihood or probability of an individual developing cardiovascular disease, a group of conditions that affect the heart and blood vessels, including but not limited to coronary artery disease, heart failure, stroke, and peripheral arterial disease. FRS has important clinical implications for early detection, prevention, and interventions to decrease the CVD burden in psychiatric patients (17). To the best of our knowledge, there is an absence of expertise evaluating the CVD risk in depressed patients with SHypo. Therefore, we aimed to compare cardiovascular risk profiles between depressed patients with or without SHypo as predicted by the FRS algorithms. We further explored the relationship between clinical variables and CVD risk. This study may identify groups at higher risk, so clinicians could use this information to consider the clinical benefit of thyroid hormone (TH) replacement therapy targeting the most vulnerable groups.

## 2. Methods

### 2.1. Participants

**Figure 1:**
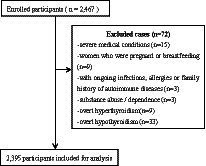
Study design flow chart.

This survey was a cross-sectional observational study design performed from 2018 to 2021. A total of 2,395 patients diagnosed with depression were recruited from the Anhui Mental Center. Two well-trained psychiatrists confirmed the diagnosis of depression using the MINI International Neuropsychiatric Interview (18) based on the ICD-10 criteria. Inclusion criteria for all participants were aged 30–74 years old, Han Chinese. No subjects had comorbidity of other psychiatric disorders, e.g., generalized anxiety and obsessive–compulsive disorders. According to these criteria, 72 patients were excluded during the enrollment: (1) severe somatic diseases (*n* = 15); (2) with ongoing infections, allergies, or exposures (*n* = 3); (3) pregnant women or women who were breastfeeding (*n* = 9); (4) substance use, abuse, and dependence (*n* = 3); (5) overt hypothyroidism (*n* = 33) and overt hyperthyroid (*n* = 9; Figure 1). All patients were taking antidepressant medication at the time of testing. All study procedures conformed to the World Medical Association Declaration of Helsinki (19) and were authorized by the Medical Ethics Review Committee of the Anhui Mental Health Center (83230230). All patients gave their informed written consent.

### 2.2. Demographic and clinical characteristics

Detailed epidemiological data of each patient were recorded on a questionnaire. Clinical information was collected from medical records. Depression severity will be assessed using the 24-item Hamilton Depression Rating Scale (HAMD-24) (20). Anxiety was measured based on the Hamilton Anxiety Rating Scale (HAMA) (21). The inter-observer correlation coefficient (ICC) for overall scores remained above 0.8 through repeated evaluation.

### 2.3. Biochemical assessment

Venous blood was collected in the morning from fasting subjects. Fasting blood glucose (FBG), high-density lipoprotein cholesterol (HDL-C), total cholesterol (TC), triglyceride (TG), low-density lipoprotein cholesterol (LDL-C), and uric acid (UA) were determined using the *Roche Cobas 8000 automatic biochemical analyzer*. The thyroid function of the enrolled patients was assessed by chemiluminescent procedures (Cobas E610; Roche, Basel, Switzerland). The laboratory reference ranges were 0.27–4.2 mIU/L for TSH, 12–22 pmol/L for FT4, and 3.1–6.8 pmol/L for FT3. Inter-assay and intra-assay variation coefficients were 5–9 or 3–6%, respectively. TSH level higher than 4.2 mIU/L with fT4 in the reference was defined as SHypo.

### 2.4. Anthropometric assessment

Weight was obtained using a calibrated digital scale nearest 0.1 kg, and height was measured to the nearest 0.1 cm using standard procedures. Body mass index (BMI) was computed as weight (kg)/height (m) squared. We employed the World Health Organization’s BMI categories, which classify BMI values as underweight (less than 18.5), normal weight (18.5–24.9), overweight (25–29.9), and obese (30 or greater). Blood pressure was measured using a standard mercury sphygmomanometer. To ensure consistency and accuracy, we followed the American Heart Association’s guidelines for defining and measuring blood pressure values in our study.

### 2.5. Assessment of cardiovascular risk

The patients underwent a medical examination including an electrocardiogram (ECG) during their hospitalization. The presence of pre-existing pathologies, such as hypertension, diabetes, dyslipidemia, or other cardiovascular diseases, was assessed by a review of medical records. The 10-year CVD risk was assessed in all patients by applying FRS. FRS was calculated based on age, gender, smoking status, TC, HDL, SBP, and history of diabetes, converted into the Framingham absolute risk, representing the probability of having a cardiovascular adverse event in the next 10 years (22).

### 2.6. Statistical analysis

Cross-comparisons were performed to calculate the 10-year CVD risk by sex group. The skewness-kurtosis test checked the normality of data distribution. Further, *t*-tests, non-parametric tests, or chi-square analyses were utilized to compare the demographic, clinical, THs, and 10-year CVD risk in both male and female patients. Continuous variables with a normal distribution are presented as the means ± SD, continuous variables with a non-normal distribution as median (interquartile range), and categorical variables are displayed as percentages. Correlation analysis was used to evaluate the association between the FRS and the clinical variables, including HAMD, HAMA, BMI, and the serum level of TT3, TT4, FT3, FT4, and TSH in both male and female patient groups separately. Further, factors significantly associated with the above in the correlation analysis were examined in multivariate regression analysis. All data analyses were performed using SPSS 19.0.

## 3. Results

### 3.1. Socio-demographic and clinical characteristics between patients with and without SHypo in male and female patients

Ultimately, 2,395 patients diagnosed with depression were included in the study, which consisted of 1,630 females and 765 males. The prevalence of SHypo in patients with depression was 11.1% (266/2,395). The prevalence of SHypo in female patients (216/1,630, 13.3%) was approximately two times that of male patients (50/765, 7.0%). In the male group, patients with SHypo had higher BMI (*t* = 2.300, *p* = 0.022), LDL-C (*t* = 7.237, *p* < 0.001), TC (*t* = 3.447, *p* = 0.001), and lower HDL-C (*t* = −4.917, *p* < 0.001) levels than patients without SHypo. In the female group, patients with SHypo had higher BMI (*t* = 2.691, *p* = 0.007), LDL-C (*t* = 6.474, *p* < 0.001), TG (*t* = 4.715, *p* < 0.001), TC (*t* = 5.782, *p* < 0.001), UA (*t* = 3.281, *p* = 0.001), lower HDL-C (*t* = −6.500, *p* < 0.001), and FT4 (*z* = −3.310, *p* = 0.001) than patients without SHypo (Table 1).

**Table 1:** Demographic and clinical characteristics of the Euthyroid-DD groups and SHypo-DD group.

	Male				Female			
	With SHypo *N* = 50	Without SHypo *N* = 715	*t*/χ2/*z*	*p* value	With SHypo *N* = 216	Without SHypo *N* = 1,414	*t*/χ2/*z*	*p* value
Age, years	55.42 ± 13.73	52.68 ± 12.03	0.874	0.386	53.97 ± 10.80	54.15 ± 11.27	−0.223	0.831
BMI (kg/m^2^)	24.131 ± 2.73	23.18 ± 2.84	2.300	0.022^*^	23.44 ± 3.23	22.85 ± 2.94	2.691	0.007^*^
Education, years	12.04 ± 3.48	11.85 ± 4.00	0.326	0.745	9.45 ± 4.15	9.55 ± 4.01	−0.356	0.722
HAMD	27.24 ± 8.40	27.60 ± 8.65	−0.287	0.774	25.10 ± 9.00	26.53 ± 9.39	−1.663	0.097
HAMA	19.49 ± 5.70	20.34 ± 7.02	−0.837	0.403	18.60 ± 7.21	19.03 ± 7.64	−0.797	0.425
SBP, mmHg	125.24 ± 12.16	126.92 ± 12.94	−0.893	0.372	123.10 ± 14.87	123.76 ± 13.54	−0.661	0.191
DBP, mmHg	80.94 ± 7.59	80.63 ± 8.85	0.239	0.811	77.38 ± 8.10	78.51 ± 8.58	−1.816	0.07
FBG (mg/dL)	5.00 ± 0.57	5.06 ± 0.80	−0.678	0.498	5.15 ± 1.22	5.08 ± 0.97	0.920	0.357
HDL-C (mmol/L)	1.11 ± 0.24	1.33 ± 0.77	−4.917	<0.001^*^	1.17 ± 0.21	1.32 ± 0.33	−6.500	<0.001^*^
LDL-C (mmol/L)	3.07 ± 0.72	2.30 ± 0.86	7.237	<0.001^*^	2.90 ± 0.80	2.54 ± 0.74	6.474	<0.001^*^
TG (mmol/L)	1.33 ± 0.71	1.45 ± 0.84	−0.579	0.489	1.76 ± 1.21	1.36 ± 0.79	4.715	<0.001^*^
TC (mmol/L)	4.75 ± 0.83	4.30 ± 0.89	3.447	0.001^*^	5.00 ± 0.96	4.61 ± 0.93	5.782	<0.001^*^
UA (μmol/L)	319.86 ± 77.22	328.71 ± 81.95	−0.740	0.439	275.64 ± 69.60	259.69 ± 66.01	3.281	0.001^*^
TT3 (nmol/L)	1.47(1.33–1.75)	1.47(1.22–1.71)	1.190	0.234	1.49(1.27–1.71)	1.44(1.18–1.68)	1.821	0.069
TT4 (nmol/L)	90.8(70.3–103.5)	87.1(64.9–101.0)	0.983	0.326	86.7(73.4–98.0)	89.3(10.9–104.0)	−1.003	0.316
FT3 (pmol/L)	4.4(3.8–5.0)	4.5(3.7–5.0)	−0.018	0.986	4.2(3.6–4.7)	4.1(3.3–4.6)	1.827	0.068
FT4 (pmol/L)	17.3(13.0–19.0)	16.5(13.1–19.3)	0.714	0.475	15.0(12.0–16.8)	16.1(1.6–18.7)	−3.310	0.001^*^
TSH (mIU/L)	5.0(4.5–6.2)	1.7(1.2–2.4)	11.833	<0.001^*^	5.9(5.0–7.8)	2.0(1.3–2.7)	23.697	<0.001^*^
10-year CVD risk (%)	10.0 (5.0–16.0)	7.0 (4.0–10.0)	4.455	<0.001^*^	8.0(5.0–11.0)	5.0(3.0–8.0)	8.722	<0.001^*^
	%	%			%	%		
Married (%)	92.0%	92.6%	0.023	0.878	92.6%	93.8%	0.440	0.507
Smoking (%)	14.0%	10.8%	0.499	0.480	0.9%	0.8%	0.052	0.820
FH (%)	24.0%	21.4%	0.187	0.665	19.4%	18.3%	0.158	0.691
Recurrence (%)	54.0%	51.5%	0.120	0.729	67.1%	61.0%	3.019	0.082

BMI, body mass index; HAMD, Hamilton depression rating scale; HAMA, Hamilton anxiety rating scale; SBP, systolic blood pressure; DBP, diastolic blood pressure; FBG, fasting blood glucose; HDL-C, high-density lipoprotein-cholesterol; LDL-C, low-density lipoprotein-cholesterol; TG, triglyceride; TC, total cholesterol; UA, uric acid; TT3, total triiodothyronine; TT4, thyroxine; FT4, free thyroxine; FT3, free triiodothyronine; TSH, thyroid-stimulating hormone; and FRS, Framingham risk score. ^*^p < 0.05.

### 3.2. Ten year CVD risk between patients with and without SHypo in male and female patients

**Figure 2:**
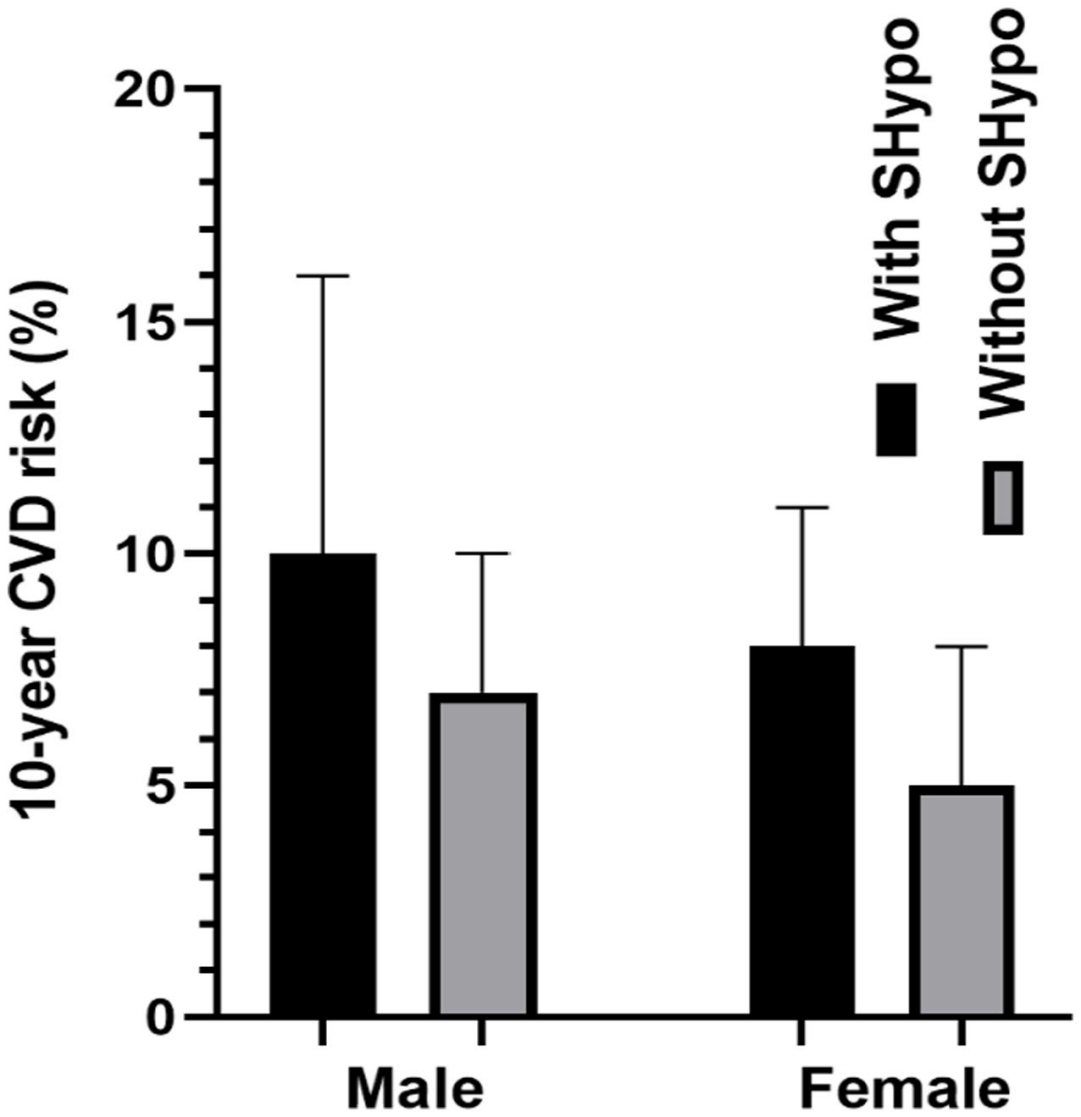
Assessment of 10-year CVD risk in female Euthyroid-DD patients and SHypo-DD patients.

Figure 1 compares the 10-year CVD risk scores in male and female patients with and without SHypo. In both male and female patients, the SHypo group had a relatively higher CVD risk than patients without SHypo (10.0 vs. 7.0%, 8.0 vs. 5.0%, *p* < 0.001; Table 1; Figure 2).

### 3.3. Association between TSH and 10-year CVD risk score in male and female patients with SHypo

Tables 2, 3 present the correlation of demographic and clinical variables in male and female patients with SHypo separately. The 10-year CVD risk score was significantly associated with BMI (*r* = 0.329, *p* < 0.001), HAMA (*r* = 0.302, *p* < 0.001), and TSH levels (*r* = 0.255, *p* < 0.001) in the female patients with SHypo (Table 3). No significant correlation was found in the male patients with SHypo (Table 4). Further, multivariable linear analysis was used to analyze the risk factors for comorbid SHypo in female patients, showing that BMI (Beta = 0.305, *t* = 5.072, *p* < 0.001), HAMA (Beta = 0.188, *t* = 4.380, *p* < 0.001), and serum TSH levels (Beta = 0.230, *t* = 3.827, *p* < 0.001) remained significant (Table 4).

**Table 2:** Correlation analysis between BMI, HAMD, HAMA, TT3, TT4, FT3, FT4, TSH, and FRS in male depressed patients with Shypo.

Variables	BMI	HAMD	HAMA	TT3	TT4	FT3	FT4	TSH	FRS
BMI	1	0.880	0.214	0.232	0.204	0.543	0.073	0.124	0.361
HAMD	0.880	1	0.001^*^	0.918	0.204	0.372	0.242	0.218	0.070
HAMA	0.214	0.001^*^	1	0.540	0.435	0.290	0.928	0.367	0.229
TT3	0.232	0.918	0.540	1	<0.001^*^	<0.001^*^	0.001^*^	0.543	0.622
TT4	0.204	0.204	0.435	<0.001^*^	1	<0.001^*^	0.001^*^	0.319	0.112
FT3	0.543	0.372	0.290	<0.001^*^	<0.001^*^	1	0.002^*^	0.071	0.063
FT4	0.073	0.242	0.928	0.001^*^	0.001^*^	0.002^*^	1	0.007	0.920
TSH	0.124	0.218	0.367	0.543	0.319	0.071	0.007^*^	1	0.897
FRS	0.361	0.070	0.229	0.622	0.112	0.063	0.920	0.897	1

^*^p < 0.05.

**Table 3:** Correlation analysis between BMI, HAMD, HAMA, TT3, TT4, FT3, FT4, TSH, and FRS in female depressed patients with Shypo.

Variables	BMI	HAMD	HAMA	TT3	TT4	FT3	FT4	TSH	FRS
BMI	1	0.839	0.260	0.080	0.151	0.542	0.938	0.343	<0.001^*^
HAMD	0.839	1	<0.001^*^	0.929	0.919	0.325	0.656	0.112	0.065
HAMA	0.260	<0.001^*^	1	0.900	0.630	0.477	0.790	0.022^*^	<0.001^*^
TT3	0.080	0.929	0.900	1	<0.001^*^	<0.001^*^	<0.001^*^	0.918	0.728
TT4	0.151	0.919	0.630	<0.001^*^	1	<0.001^*^	0.001^*^	0.343	0.660
FT3	0.542	0.325	0.477	<0.001^*^	<0.001^*^	1	<0.001^*^	0.727	0.160
FT4	0.938	0.656	0.790	<0.001^*^	0.001^*^	<0.001^*^	1	0.235	0.273
TSH	0.343	0.112	0.022^*^	0.918	0.343	0.727	0.235	1	<0.001^*^
FRS	<0.001^*^	0.065	<0.001^*^	0.728	0.660	0.160	0.273	<0.001^*^	1

^*^p < 0.05.

**Table 4:** Hierarchical regression analysis on Framingham risk scores for female SHypo-DD patients.

	Coefficients		Beta	*t*	*p* value	95% confidence interval for EXP	
	B	Std. error				Lower	Upper
(Constant)	−9.018	2.422		−3.723	<0.001	−13.792	−4.244
BMI	0.485	0.096	0.305	5.072	<0.001^*^	0.296	0.673
HAMA	0.188	0.043	0.264	4.380	<0.001^*^	0.103	0.273
TSH	0.402	0.105	0.230	3.827	<0.001^*^	0.195	0.610

^*^p < 0.05.

## 4. Discussion

Our study aimed to investigate the prevalence of SHypo in patients with depression and its impact on their 10-year cardiovascular disease (CVD) risk. The results showed that 11.1% of depressed patients had SHypo, with a higher prevalence in females than males. Patients with comorbid SHypo had a higher 10-year CVD risk than those without SHypo. In female patients with SHypo, serum TSH levels, BMI, and anxiety symptoms were associated with increased 10-year CVD risk. These findings highlight the need for early detection and management of SHypo in depressed patients, especially in females, to prevent CVD complications.

Our study suggested that the prevalence of SHypo is 11.1% in patients with depression, which is consistent with our previous study (23). According to the TRH hypothesis of depression (24), chronic TRH hypersecretion in depression can be a compensatory mechanism to normalize serotonin (5-HT) function (25). As a result, elevated TSH levels are often found among depressed patients, possibly increasing the chances of developing SHypo. Furthermore, our study showed that depressed patients with comorbid SHypo exhibited a higher 10-year CVD risk than patients without SHypo in both males and females. One possible explanation is that patients with SHypo harbor unfavorable changes in several metabolic parameters, including lipid profiles and glucose homeostasis, which may amplify the CVD risk (26–28). A recent study also found that depressed individuals with SHypo are more likely to meet the criteria for metabolic syndrome (MetS), which was a significant risk factor for CVD (29). In the current study, all male and female patients with SHypo demonstrated significantly higher levels of TC, UA, LDL-C, and lower levels of HDL-C than euthyroid patients. These results further highlight that depressed patients with SHypo may have a higher burden of lipid metabolic disorder, likely contributing to CVD risk.

Our study found a remarkable association between TSH levels and 10-year CVD risk in female depressed patients with comorbid SHypo, following former knowledge highlighting that SHypo may have favorable effects on CVD through the TSH effect. Previous studies have identified a positive relationship between TSH levels and lipid profiles. One study found a 2-fold increased risk of metabolic syndrome in women with TSH levels >2.5 mIU/L relatives compared to those with TSH levels <2.5 mIU/L (30, 31). Another population-based study including 30,656 individuals demonstrated that deranged lipid concentrations were linearly associated with increasing levels of TSH across the entire reference range (32). In addition, a recent study also found that TSH levels were positively associated with 10-year CVD risk scores, especially in females (30). Other recent studies also suggested that high TSH concentrations are related to increased CVD risk, demonstrating that variations in the normative range of TSH should be considered when assessing long-term adverse health outcomes (2, 33). It is widely accepted that TH plays a crucial role in glucose and lipid homeostasis and regulates heart function and the peripheral vascular system (34). By influencing calcium uptake and altering adrenergic and cholinergic receptors sodium-potassium adenosine triphosphatase (ATPase), insufficient TH concentrations can impair the relaxation of vascular smooth muscle cells and inhibit cardiac contractility (35, 36). Low TH levels possibly contribute to the increase in systematic vascular resistance and cause endothelial changes by decreasing nitric oxide availability (37). Therefore, it was suggested that thyroid hormone administration in SHypo subjects could improve serum lipid profile and other cardiovascular risk factors, thereby decreasing cardiovascular risks (38, 39).

To the best of our knowledge, this is the first study to investigate the relationship between anxiety, thyroid hormones, and CVD risk, as previous studies have primarily focused on either psychological factors or thyroid hormones independently (2, 40, 41). As discussed in our previous study, female patients with depression exhibited higher anxiety/somatization factor scores than males (42). The current study also found that HAMA scores were associated with the increased 10-year CVD risk. Notably, a consistent body of literature demonstrated anxiety as a risk factor for cardiovascular disease independent of depression (16, 17). A meta-analysis also showed that anxiety was related to an increased risk for CVD (43). Another two studies have examined the association of anxiety with CVD risk, identifying a significant increase in CVD risk (44, 45). Considering the possible biological mechanism linking anxiety to higher CVD risk, anxiety may lead to hyperactivation of the hypothalamic–pituitary–adrenal (HPA) axis and sympathetic nervous system and increase plasma catecholamines, further damage the vascular endothelium and increase CVD risk (46).

Anxiety and depression are common comorbidities, and both have been associated with an increased risk of CVD. Anxiety can heighten an individual’s cardiovascular responses to stress, raise resting heart rate, and induce dysfunction of arterial baroreflex, finally leading to an increase in cardiac workload (47). Moreover, a recent observational study demonstrated that anxiety was related to elevated TSH levels in patients with depression (15). Therefore, the coexistence of subclinical hypothyroidism and depression may worsen anxiety symptoms and increase the 10-year CVD risk, which warrants further attention by clinicians and researchers.

Our study also revealed that depressed patients with SHypo had slightly higher BMI than euthyroid patients. Moreover, increasing BMI was confirmed to be independently associated with 10-year CVD risk in female depressed patients with SHypo. It has been reported that serum TSH levels were positively related to increasing BMI (48). One previous study also noted the correlation between SHypo and higher BMI in women but not men (49). It has been suggested that depressed individuals may try to cope with stress and relieve depression by increasing their eating behavior, leading to an increased risk of overweight/obesity (50). Numerous studies have confirmed that excess weight and obesity increase the risk of developing cardiovascular diseases (51, 52). Therefore, low BMI or body weight was critical to preventing cardiovascular disease in female depressed patients with SHypo.

Our study provides novel insights into the association between SHypo and CVD risk in patients with depression. Our findings are consistent with previous studies showing that SHypo is a risk factor for CVD in the general population (53, 54). However, our study is the first to investigate this association in patients with depression, a high-risk group for CVD. Importantly, we found that the association between SHypo and CVD risk was stronger in women than in men, which suggests that gender may play a role in this association. The gender differences in the association between SHypo and CVD risk have important clinical implications. Our findings suggest that clinicians should pay particular attention to SHypo in women with depression, as they may be at higher risk for CVD. The mechanisms underlying this gender difference are not clear and warrant further investigation. One possibility is that sex hormones may play a role, as estrogen has been shown to have protective effects on the cardiovascular system (55). Further research is needed to explore this hypothesis.

This work is subject to some limitations. Firstly, given the cross-sectional design, we could not infer causality. Secondly, this was a convenience sample recruited from a simple hospital. Therefore, it might be hard to generalize our findings to other groups. Thirdly, it is difficult to ascertain whether SHypo occurs before or after depression because the baseline thyroid function was unknown. Fourthly, there were no healthy controls in the present study. Fifthly, we did not collect comprehensive information on pre-existing pathologies or other risk factors that could affect the patient’s cardiovascular risk profile. Therefore, we cannot exclude the possibility that these factors may have influenced our results. Sixthly, while our study is limited to patients with depression, these findings provide insight into the potential link between anxiety, thyroid hormones, and cardiovascular disease risk. Future studies should aim to replicate these findings in a larger, more diverse population to understand our results’ generalizability better.

## 5. Conclusion

We found an increased 10-year CVD risk in depressed patients with comorbid SHypo. Our study also highlights the importance of considering gender differences in this association, as we found that serum TSH levels, BMI, and anxiety symptoms were associated with increased 10-year CVD risk in female depressed patients with comorbid SHypo. However, further studies with a larger sample size are needed to confirm these findings. Clinicians need to be aware of the need to regularly monitor thyroid function in depressed patients, particularly in females with comorbid SHypo who may be at higher risk of CVD. Further prospective studies are required to determine whether thyroid hormone supplementation has beneficial effects in reducing cardiovascular risk.

## Data availability statement

The original contributions presented in the study are included in the article/supplementary material, further inquiries can be directed to the corresponding author.

## Ethics statement

The studies involving human participants were reviewed and approved by Medical Ethics Review Committee of the Anhui Mental Health Center. The patients/participants provided their written informed consent to participate in this study.

## Author contributions

SZ and AW: conceptualization, methodology, software, investigation, formal analysis, and writing—original draft. BZ and YH: data curation and writing—original draft. JG and WF: software and validation. HZ: visualization and writing—review and editing. All authors contributed to the article and approved the submitted version.

## Funding

This study was supported by grants of Anhui Provincial Medical and Health Key Specialties Project.

## Conflict of interest

The authors declare that the research was conducted in the absence of any commercial or financial relationships that could be construed as a potential conflict of interest.

## Publisher’s note

All claims expressed in this article are solely those of the authors and do not necessarily represent those of their affiliated organizations, or those of the publisher, the editors and the reviewers. Any product that may be evaluated in this article, or claim that may be made by its manufacturer, is not guaranteed or endorsed by the publisher.
